# Health and health needs of migrants in detention in Greece: shedding light to an unknown reality

**DOI:** 10.1186/s12992-018-0448-4

**Published:** 2019-01-08

**Authors:** Kyriakos Souliotis, Maria Saridi, Konstantina Banou, Christina Golna, Dimitrios Paraskevis, Angelos Hatzakis, Alyna Smith

**Affiliations:** 1 Faculty of Social and Political Sciences, University of Peloponnese, Corinth, Greece; 2 Health Policy Institute, Athens, Greece; 3 Policy Change, Innowth Ltd, Larnaca, Cyprus; 4 Department of Hygiene, Epidemiology and Medical Statistics, Medical School, National and Kapodistrian University of Athens, Athens, Greece; 5 PICUM - Platform for International Cooperation on Undocumented Migrants, Brussels, Belgium

**Keywords:** Migrants, Public health, Detention centres, Screening, Surveillance

## Abstract

**Background:**

Population movements have been increasing over the past years in Europe due to socioeconomic factors, global turbulence and conflicts, especially in the area of Middle East. The presence of migrant populations in Europe challenges health systems due to increased requirements for health care provision. However, to date there is limited published data on the burden of disease among this population (in Greece and elsewhere). Our objective was to record burden of disease of undocumented migrants hosted in a Detention Center and therefore generate data for migrant and public health planning.

**Methods:**

Epidemiological data have been collected for 4756 male migrants hosted in a Detention Center from mid 2013 to mid 2015. Of them, 1427 have used health services in the Center, which maintained a detailed record of their medical history and tests.

**Results:**

The majority of the study population was aged between 18 and 40 years old. Among those who used health services, most suffered from respiratory (45.6%) and digestive (30.1%) diseases. Injury, poisoning and other external causes accounted for 19.6% of service use, diseases of the skin and subcutaneous tissue for 18.7%, and factors affecting health status and contact with health services for 16.7%. Prevalence of communicable diseases was 15.9% amongst migrants randomly tested.

**Conclusion:**

Systematic screening and monitoring of diseases and use of health services by migrants in detention centers allows for an evidence based understanding of the burden of disease related to these populations and the investment required to effectively manage it, thus providing critical input to appropriate health planning. Surveillance for communicable diseases amongst migrants in detention centers would also allow for a true picture of the impact of their presence on public health indicators and help address related prejudices and stigma.

## Background

Global conflict, poverty and economic inequalities have led to a dramatic increase in the numbers of internally displaced persons and refugees worldwide. Europe due to its geographic location at the cross-road of three continents has been a target host continent, receiving over 1 million refugees or migrants/immigrants via the Mediterranean in 2015. Almost a third of them were children according to the United Nations High Commissioner for Refugees [1, 2]. About 1.2 million applied for asylum in European Union (EU) countries in 2015, more than twice the number in 2014 [3].

Both documented and undocumented migrants are recognized as a group facing numerous barriers to accessing adequate health services in host countries [4, 5], due to, among others, language, marginalization, bureaucracy, and fear of deportation. These hold true in Greece, where a previous study confirmed cultural and language barriers to accessing mental health services by undocumented migrants [6]. Papadakaki et al. [7] reported on the outcomes of the RESTORE project in Crete, Greece, where health care providers mentioned feeling powerless about supporting migrant healthcare with such low capacity in the system, due to primarily fiscal constraints. They also emphasized lack of training and skills for working in cross-cultural consultations. Provider level barriers also optated at the structural level including lack of interpreter services, access to primary care appointments, lack of access to health insurance [8]. The latter is also highlighted in a very recent review [9]. Access to healthcare is an important part of the humanitarian response to the immigration crisis and is a recognized human right [10]. Epidemiological or clinical data of such vulnerable populations could be used to guide screening and inform efficient health planning that would adequately address the population’s needs. Such data could equally help draw an objective picture of migrant health status, health needs and health service use, thus helping address related prejudices and stigma.

To date, the limited literature published on migrant health does not allow for a proper understanding of the actual burden of disease associated with migrants or their health needs [11]. This further hinders the development of appropriate hosting policies and structures that would help make the transition for these populations smoother.

This study reports on the burden of disease of undocumented migrants hosted in a Detention Center in Greece. The use of the term migrant refers to people on a continuous move, with an unknown status of residence. To our knowledge this is the only study describing the epidemiological status of migrants in Detention Centers in Greece.

## Population and methods

Study population included 4756 migrants held in the Detention Center of Corinth between mid 2013 and mid 2015. The center was male only, including boys and was selected because of its capacity to host large numbers of migrants and provide health care services to them. Study sample included all migrants who made any use of health services in the Detention Center, including day use. Additional visits were recorded at the clinic of the Centre, where patients were examined by staff supervised by the Hellenic Center for Disease Control and Prevention (HCDCP), and by the police force responsible for monitoring psychiatric cases. Excluded were migrants who lacked identity data (such as nationality or age) and migrants whose medical records were not readable or could not be classified according to the International Statistical Classification of Diseases (ICD-10). The study was performed on medical records maintained in the Center, access to which was authorized by the Chief of the Hellenic Police Force. All patient data were encoded for confidentiality purposes.

## Results

**Fig. 1:**
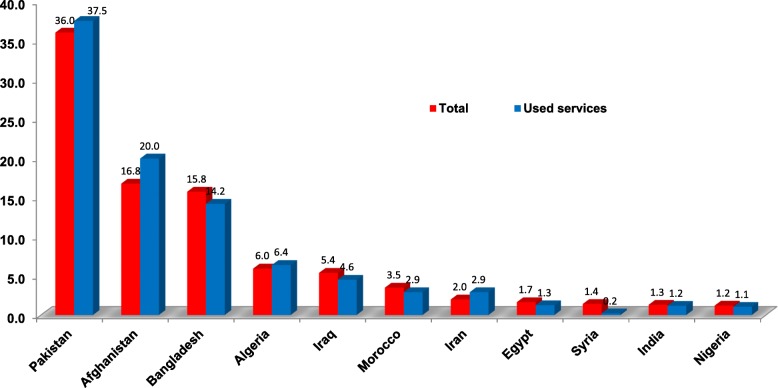
Percentage of migrant population (total and service use) in Detention Center, by country of origin

The majority of migrants were from Asia (80.1%), most commonly from Pakistan (36%), Afghanistan (15.8%) and Bangladesh (15.8%) (Table 1). Their age ranged mostly between 18 to 30 years old (68.9% of total). 22 of the total sample (0.5%) were minors. Of the total (*n* = 4756), 1427 (30%) made any use of health services. Their nationality is depicted on Table 2. The percentage of migrants who used health services per country of origin was proportionate to the percentage of migrants per country of origin in the Center (Fig. 1).

**Table 1:** Total migrant population in Detention Center, by country of origin

Geographic area											
Africa (1)			Africa (2)			Asia			Europe		
Country	*N*	(%)	Country	*N*	(%)	Country	*N*	(%)	Country	*N*	(%)
Algeria	285	5.99	Mali	7	0.15	Pakistan	1712	36.00	Georgia	18	0.38
Morocco	168	3.53	Libya	6	0.13	Afghanistan	799	16.80	Albania	3	0.06
Egypt	79	1.66	Sierra Leone	6	0.13	Bangladesh	750	15.77	Bosnia	1	0.02
Nigeria	58	1.22	Mauritania	4	0.08	Iraq	259	5.45	Ukraine	1	0.02
Tunisia	46	0.97	Tanzania	4	0.08	Iran	96	2.02	Poland	1	0.02
Sudan	43	0.90	Angola	2	0.04	Syria	68	1.43	Total Europe	24	0.50
Eritrea	35	0.74	Ethiopia	2	0.04	India	63	1.32			
Senegal	32	0.67	Cameroon	2	0.04	Palestine	31	0.65	America		
Somalia	27	0.57	Togo	2	0.04	Nepal	6	0.13	Dominican Republic	2	0.04
Congo	17	0.36	Liberia	1	0.02	China	5	0.11	Jamaica	2	0.04
Ivory Coast	14	0.29	Burkina Faso	1	0.02	Turkey	5	0.11	Haiti	1	0.02
Ghana	11	0.23	Uganda	1	0.02	Myanmar	3	0.06	Total America	5	0.11
Guinea	9	0.19	Rwanda	1	0.02	Sri Lanka	3	0.06			
Comoros	9	0.19			18.48	Lao	2	0.04			
Gambia	7	0.15			0.15	Lebanon	2	0.04	Unspecified	40	0.84
					0.13	Philippines	2	0.04			
Total Africa	879	18.48				Mongolia	1	0.02			
						Yemen	1	0.02			
						Total Asia	3808	80.07			
Total	4756	100.00

**Table 2:** Migrant population in Detention Center, who made at least one use of health services, by country of origin

Geographic area											
Africa (1)			Africa (2)			Asia			Europe		
Country	*N*	(%)	Country	*N*	(%)	Country	N	(%)	Country	*N*	(%)
Algeria	92	6.45	Sierra Leone	3	0.21	Pakistan	535	37.49	Georgia	3	0.21
Morocco	42	2.94	Somalia	3	0.21	Afghanistan	285	19.97	Bosnia	1	0.07
Egypt	18	1.26	Gambia	2	0.14	Bangladesh	203	14.23			
Nigeria	15	1.05	Eritrea	2	0.14	Iraq	65	4.56	Total Europe	4	0.28
Tunisia	15	1.05	Congo	2	0.14	Iran	42	2.94			
Senegal	12	0.84	Mali	2	0.14	India	17	1.19			
Sudan	9	0.63	Ethiopia	1	0.07	Palestine	13	0.91	Unspecified	18	1.26
Ivory Coast	4	0.28	Cameroon	1	0.07	Syria	3	0.21			
Ghana	3	0.21	Comoros	1	0.07	Turkey	3	0.21			
Guinea	3	0.21	Mauritania	1	0.07	China	2	0.14			
Libya	3	0.21	Tanzania	1	0.07	Myanmar	1	0.07			
						Philippines	1	0.07			
Total Africa	235	16.47				Total Asia	1170	81.99			
Total	1427	100.00

Table 3 depicts prevalence of disease by ICD-10 code among study sample (*n* = 1427). Some migrants made use of health services for more than one condition. The majority (45.6%) suffered from respiratory diseases (acute upper and lower respiratory infection, common cold, etc.), while 30.1% were diagnosed with digestive diseases (teeth and supporting structures related disorders, gastritis and constipation) and 24.9% had symptoms that fell under the R00 classification (not classified elsewhere) in ICD-10. Injury, poisoning and certain other external causes accounted for 19.6% of total disease prevalence, while diseases of skin and subcutaneous tissue for 18.7%. The prevalence of communicable diseases (tuberculosis, hepatitis, etc.) was 16%, and patients were either referred to a hospital for observation or directly examined at the hospital of referral. Additionally, 12.8% of total suffered from acute nasopharyngitis, 10.7% from diseases of the teeth and their supporting tissues, 9.7% had abdominal and pelvic pain, 9.4% suffered from diseases of the lower respiratory tract and 8.7% had various forms of dermatitis.

**Table 3:** Diagnoses as a percentage of total, migrants who used health services, by ICD-10

Classification ICD 10		N	%
A00-B99	Certain infectious and parasitic diseases	227	15.9
D50-D89	Blood diseases of blood-forming organs and certain disorders of the immune mechanism	3	0.2
E00-E90	Endocrine, nutritional and metabolic disorders	18	1.3
F00-F99	Mental and behavioral disorders	147	10.3
G00-G99	Diseases of the nervous system	31	2.2
H00-H59	Diseases of eye and adnexa	103	7.2
H60-H95	Diseases of the ear and mastoid process	57	4.0
I00-I99	Diseases of the circulatory system	39	2.7
J00-J99	Diseases of the respiratory system	650	45.6
K00-K93	Digestive diseases	429	30.1
L00-L99	Diseases of the skin and subcutaneous tissue	267	18.7
M00-M99	Diseases of the musculoskeletal system and the connective tissue	222	15.6
N00-N99	Diseases of the genitourinary system	100	7.0
Q00-Q99	Congenital disorders, malformations and chromosomal abnormalities	3	0.2
R00-R99	Symptoms, signs and abnormal clinical and laboratory findings, not classified	356	24.9
S00-T98	Consequences of injury. Poisoning and other external causes	279	19.6
V01-Y98	External causes of morbidity and mortality	62	4.3
Z00-Z99	Factors influencing health status and contact with health services	239	16.7
AF	Referring hemoptysis	13	0.9
AA	Attempted suicide	16	1.1
AP	Hunger strike	9	0.6
ΠΤ	Wound care	49	3.4
ΜΝ	Solitary kidney	3	0.2
NONE		23	1.6

Among those who used health services (*n* = 1427), 7.3% (*n* = 104) underwent mantoux examination. 28 of them (26.9% of tested migrants or 2% of migrants who used health services) tested positive. Among the 28 migrants who tested positive, one person was hospitalized to investigate a positive result, one person reported previous history of tuberculosis, and six people were put on prophylactic treatment for six months. The majority of migrants (42.9%) who tested positive were from Pakistan. Additionally, 13 migrants were recorded with hemoptysis, the majority (6) from Pakistan.

Among the 80 migrants who tested in random for communicable diseases, 13 (16.5%) were diagnosed with at least one type of viral hepatitis. 3 people were found to have a solitary kidney of unknown etiology.

**Fig. 2:**
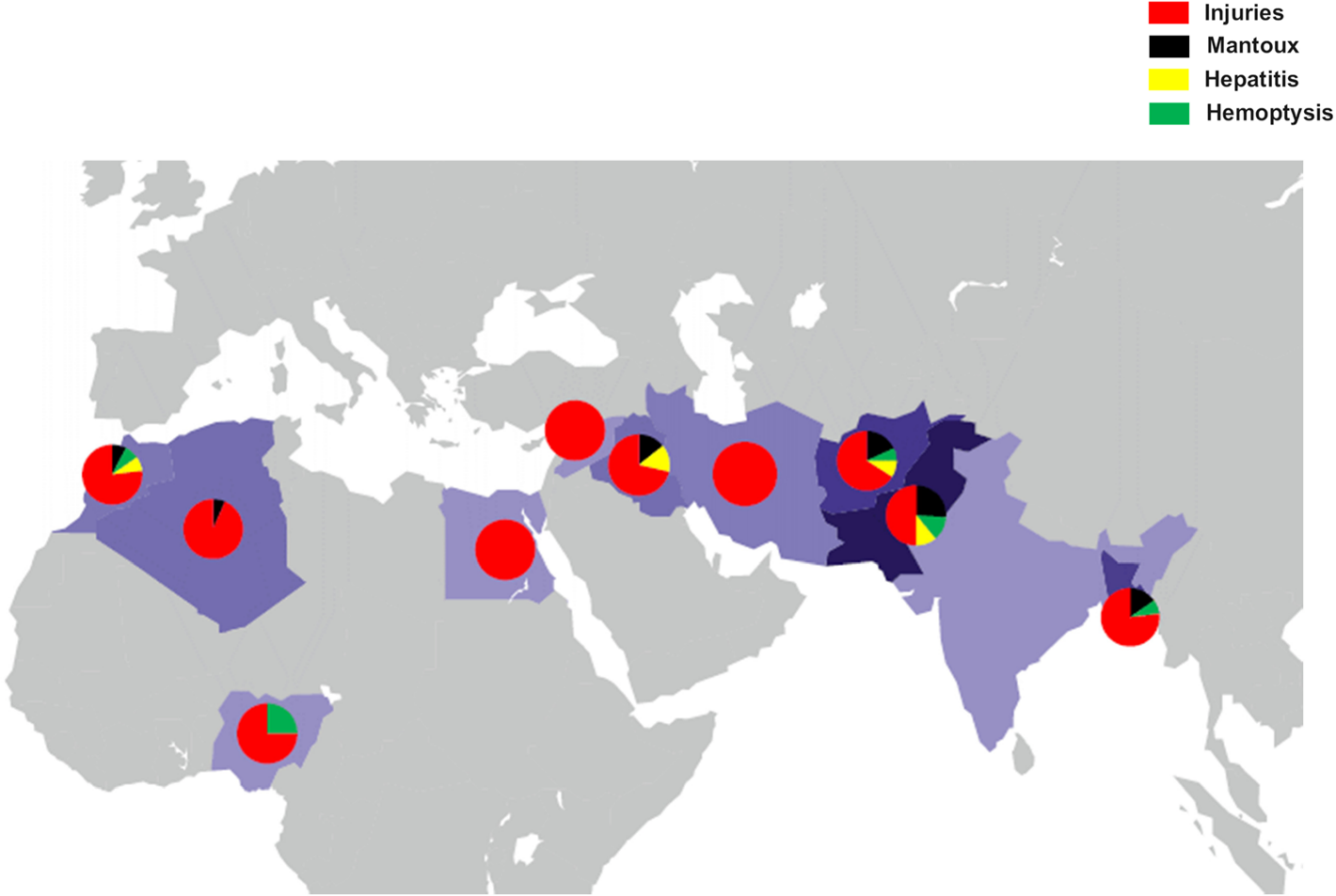
Countries of origin for select diagnoses, migrants who used health services. Note: Percentage of migrant population per country of origin that used health services for (**a**) injuries (red color), (**b**) mantoux (black color), (**c**) hepatitis (yellow color) and (**d**) hemoptysis (green color)

Figure 2 depicts country of origin of migrants who used health services for injuries, mantoux, hepatitis and hemoptysis.

16 people attempted suicide. This number excludes people who attempted suicide (e.g. people climbed to a high point and threatened to jump) and changed their minds. The latter were referred to psychologists in the Center or psychiatrists in the General Hospital of Corinth. 31.3% were from Algeria, 25% from Palestine and 75% of them were between 18 to 30 years old.

## Discussion

Greece has become one of the main entry points for irregular arrivals to Europe in the recent years, the vast majority of them being migrants and refugees. Their health is equally becoming a central element of social cohesion in western societies. Understanding migrant health needs allows for adequate planning and investment of resources, increased integration of migrant with mainstream health services and improved public health outcomes – to the benefit of not only migrants but also host societies.

Our study aimed to shed light on the epidemiological profile and the health needs of migrants staying in a Detention Center in Greece, in order to help address those needs as well as fight any stigma that may be attached to these populations, because of their “presumed” health burden.

Findings from our study are consistent with previous studies, regarding the sex, age distribution and health status of migrants. [12, 13]. Migrants are deemed to be physically robust and relatively young in age. The majority of migrants in our study were between 18 and 30 years old, followed by the 31–40 years old age group.

Yet, our study findings cannot confirm nor refute previous literature on the high prevalence of communicable diseases amongst migrants [14–16]. For instance, our findings of positive mantoux among our tested study sample (2% of those who used health services, which in itself may be a small sample to allow for generalisation) cannot be construed to indicate presence of active tuberculosis – this has been confirmed only in one case referred for hospitalisation. This is in line with findings from a study conducted at the Greek-Turkish border migrant camp [13]. In any case, living conditions in overcrowded camps may favor both the transmission and the occurrence of the disease. In a study in Spain, a tuberculin skin test was performed on 230 asymptomatic patients, latent tuberculosis infection was diagnosed in 100 (43.5%) patients, and 4 patients were diagnosed with active tuberculosis [17]. Further, the diagnosis of 13 cases of viral hepatitis (of which 7 were HBV cases) amongst the 80 migrants randomly tested for communicable diseases cannot confirm nor refute previous findings on the resurgence of viral hepatitis, including HBV, in Europe, due to increased migration [18–20]. Again, this finding is in line with a previous study specific to migrant camps in Greece [13].

Our study confirmed a large number of injuries (almost 1 out of 5) amongst those who requested health services within the Detention Center. This is a worrying finding, as it depicts an unsafe detention environment, despite constant police presence. It may also account in some part for the relatively high prevalence of psychiatric conditions, amongst which fear, anxiety and depression rate high. Such factors may also be affected by the duration of stay in detention – some of these migrants may face extended stays in detention until either their papers or their exportation is processed. Recorded attempted suicide rate (16 cases) is a confirmation of the stress (post traumatic and other) migrants in detention centers are under and is in line with recent European studies of asylum seekers, which show increased rates of suicide, especially among male asylum seekers, and increased suicidal behavior compared with the general population [21, 22]. Stress is also due to the transition: people who come to Europe may have experienced very different health systems in their countries of origin, and face challenges navigating a new and unfamiliar health system. Some face additional barriers, because of laws that limit their ability to access health care, because of their residence status, or because they are in detention. Detention is in itself both a cause of diminished physical and mental health, and a place where access to adequate health care is generally limited. The result is a double blow to the health of people in detention [23, 24].

## Conclusion

Our study findings are to be reached with a pinch of salt: a male only sampling, lack of organized screening, random testing in very limited numbers of migrants and lack of systematic recording of epidemiological profiles and health needs allow for a critical opportunity to correctly map the health and health needs of such populations to be missed. They also limit the findings of any such study that aims to provide a basis for understanding and discussion on migrant health and health needs. They equally open the back door to stigma and prejudice and undermine future integration of such populations in host societies.

And all this despite significant availability of funding for structured interventions: Greece has been on the receiving end of substantial funds from international and European donors to set up and manage detention centers and cater to the needs of migrants. It is a rare case of funds being available to invest in improving disease surveillance and mapping, understanding and informing migrant policies, in Greece and beyond, and a great misfortune if such an opportunity is to be lost because of lack of planning and implementation management.

## Abbreviations

HBV: Hepatitis B virus
HCDCP: Hellenic Center for Disease Control and Prevention
ICD-10: International Statistical Classification of Diseases
